# Discontinuation of Hemodialysis in a Patient with Anti-GBM Disease by the Treatment with Corticosteroids and Plasmapheresis despite Several Predictors for Dialysis-Dependence

**DOI:** 10.1155/2017/7143649

**Published:** 2017-10-11

**Authors:** Yoshihide Fujigaki, Chikayuki Morimoto, Risa Iino, Kei Taniguchi, Yosuke Kawamorita, Shinichiro Asakawa, Daigo Toyoki, Shinako Miyano, Wataru Fujii, Tatsuru Ota, Shigeru Shibata, Shunya Uchida

**Affiliations:** ^1^Department of Internal Medicine, Teikyo University School of Medicine, Itabashi-ku, Tokyo, Japan; ^2^Central Laboratory, Teikyo University School of Medicine, Itabashi-ku, Tokyo, Japan

## Abstract

A 26-year-old man highly suspected of having antiglomerular basement membrane (GBM) disease was treated with corticosteroid pulse therapy 9 days after initial infection-like symptoms with high procalcitonin value. The patient required hemodialysis the next day of the treatment due to oliguria. In addition to corticosteroid therapy, plasmapheresis was introduced and the patient could discontinue hemodialysis 43 days after the treatment. Kidney biopsy after initiation of hemodialysis confirmed anti-GBM disease with 86.3% crescent formation. Physician should keep in mind that active anti-GBM disease shows even high procalcitonin value in the absence of infection. To pursue recovery of renal function, the challenge of the immediate and persistent treatment with high-dose corticosteroids plus plasmapheresis for highly suspected anti-GBM disease is vitally important despite the presence of reported predictors for dialysis-dependence including oliguria and requiring hemodialysis at presentation.

## 1. Introduction

Antiglomerular basement membrane (GBM) disease is rare condition usually with rapidly progressive glomerulonephritis (RPGN) and is known as Goodpasture's syndrome when it combines with pulmonary hemorrhage [1]. All patients with anti-GBM disease have circulating and deposited anti-GBM antibody. An early aggressive immunosuppressive treatment to inhibit production of anti-GBM antibody and/or attenuating the antibody-mediated glomerular inflammation and plasmapheresis to reduce or remove anti-GBM antibody are necessary [2, 3], as a recovery of renal function rarely occurs in patients with advanced stage where predictors of dialysis-dependence such as oliguria, requiring hemodialysis and high percentage of crescent formation, were seen [4–6]. However, the patients with anti-GBM disease with severe renal dysfunction are immunocompromised and often show fever, positive C-reactive protein (CRP), and positive procalcitonin (PCT) [7, 8] as an indicator of infection. These factors usually become an obstacle in an early aggressive immunosuppressive therapy.

We present a patient with anti-GBM disease who showed high PCT value and all reported predictors for dialysis-dependence but could discontinue hemodialysis by introducing immunosuppressive therapy plus plasmapheresis. In this case, we add some personal opinions to the recommendation for anti-GBM disease in Kidney Disease Improving Global Outcomes (KDIGO) clinical practice guideline for glomerulonephritis [9].

## 2. Clinical Presentation

A 26-year-old Japanese man visited a clinic complaining about fever and coral-colored urine 7 days previously. Antihypertensive drug and antibiotics were prescribed for high blood pressure and suspected urinary tract infection, respectively. He was admitted to local hospital 4 days previously because of high fever, general fatigue, nausea, abnormal urinalysis (2+ protein and 3+ occult blood), renal dysfunction (serum creatinine (SCr) of 1.66 mg/dl), and C-reactive protein (CRP) of 15 mg/dl. Since SCr was further increased to 3.39 mg/dl, he was transferred to our hospital.

On admission, he showed body temperature of 37.9°C, blood pressure of 142/84 mmHg, pulse rate of 101/minute, and SpO_2_ of 98% and no other abnormal physical examination. He had no smoking and past medical history. Anti-GBM antibody examined in the previous hospital was reported to be positive. Laboratory examination showed proteinuria, hematuria, SCr of 4.49 mg/dl, CRP of 21.04 mg/dl, PCT of 0.62 ng/dl (normal range < 0.05), normocomplementemia, and anti-GBM antibody of 350.0 U/ml (normal range < 3). Myeloperoxidase- and proteinase 3-antineutrophil cytoplasmic antibodies, other autoantibodies, and cryoglobulin were negative. Chest X-ray did not show pulmonary hemorrhage. He was diagnosed as RPGN most likely due to anti-GBM disease. He was given antibiotics after hospitalization for 1 week because both CRP and PCT were high with fever, but he had no apparent focus of infection.

**Figure 1:**
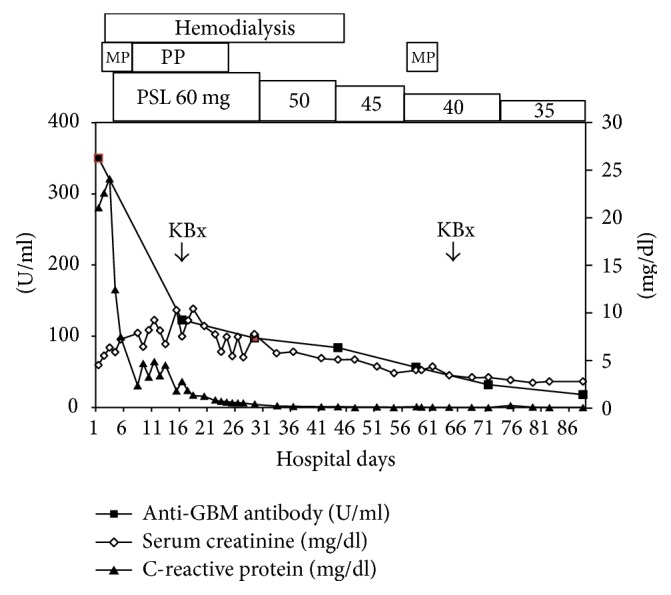
Clinical course. MP, methylprednisolone pulse therapy (1 g per day for 3 successive days); PP, plasmapheresis 7 times; PSL, prednisolone; KBx, kidney biopsy.

Clinical course after admission was shown in Figure 1. The patient was treated with methylprednisolone pulse therapy (1 g per day for 3 successive days) from 2nd day after admission and then with oral prednisolone 60 mg/day. Coral-color urine and high-grade fever disappeared at 3rd day. However, he was introduced to hemodialysis at 3rd day because of oliguria, further increased SCr of 6.3 mg/dl, severe metabolic acidosis, and hyperkalemia. Plasmapheresis (3,840 ml of plasma with fresh frozen plasma of 32 units as the substitution) through the polyethylene plasma separator OP-05W (Asahi Kasei Medical, Co., Ltd., Tokyo, Japan) began from 5th day seven times.

**Figure 2:**
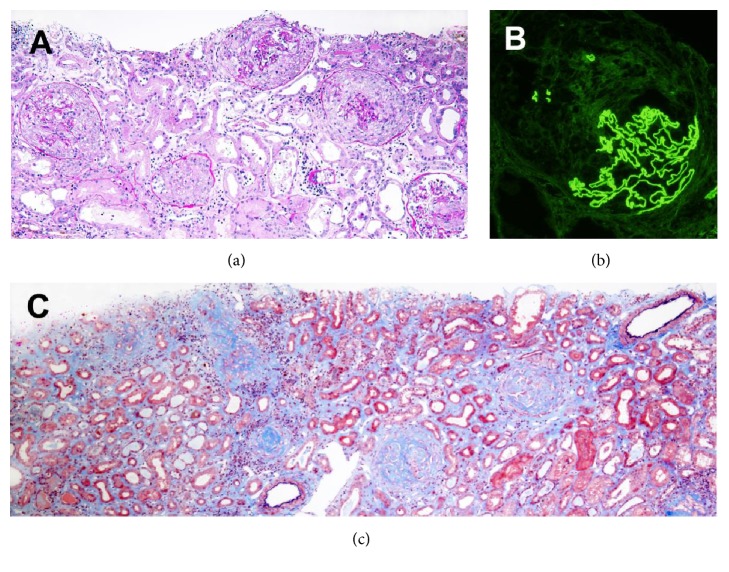
Light microscopic findings of 1st (a and b) and 2nd (c) kidney biopsies. (a) There are glomeruli demonstrating cellular or fibrocellular crescents with or without focal segmental necrosis and diffuse inflammatory cell infiltration, patchy tubular injury, and mild fibrosis in the tubulointerstitial areas (PAS staining, ×200). (b) Immunofluorescent staining for IgG shows linear staining along with glomerular capillary walls, ×400. (c) There are globally sclerotic glomeruli and diffuse tubulointerstitial fibrosis with mononuclear cell infiltration and tubular atrophy (Elastica-Masson staining, ×200).

Kidney biopsy at 16th day showed cellular or fibrocellular crescents with or without necrotic glomerular capillary walls in 19 of 22 glomeruli (86.3%) (Figure 2(a)). There were diffuse inflammatory cell infiltration, patchy tubular injury, and mild fibrosis in the tubulointerstitial areas (Figure 2(a)). Immunofluorescence for IgG showed 2+ linear staining along the glomerular capillary walls (Figure 2(b)). The findings confirmed anti-GBM disease.

Blood pressure during the hemodialysis period was around 150/80 mmHg using doxazosin mesilate. Under high-dose prednisolone urine volume began to increase at 30th day and he could discontinue hemodialysis at 44th day. Methylprednisolone pulse therapy was added (1 g per day for 3 successive days) from 57th day because of the presence of active urinalysis and anti-GBM antibody of 56.6 U/ml. The second kidney biopsy at 65th day revealed 16 glomeruli with global sclerosis and 7 glomeruli with fibrous crescents out of 24 glomeruli and diffuse tubulointerstitial fibrosis with mononuclear cell infiltration and tubular atrophy (Figure 2(c)), indicating no active glomerular lesions. Prednisolone was tapered to 35 mg/day and he was discharged with SCr of 2.74 mg/dl and anti-GBM antibody of 18.2 U/ml at 88th day.

After the discharge, blood pressure had been controlled at around 140/80 mmHg with doxazosin mesilate and nifedipine. 1.0 to 1.5 g/gCr of proteinuria and mild degree of hematuria persisted. Estimated glomerular filtration rate (eGFR) was stable at about 25 ml/min/1.73 m^2^ for 4 months after the discharge. However, eGFR began to decrease after that and anti-GBM antibody titer slightly rose; thus methylprednisolone pulse therapy was performed again. Anti-GBM antibody became negative at the dose of 30 mg/day of prednisolone, which was further tapered. The patient did not have any side effects during the immunosuppressive therapy. However, eGFR decline slope was almost constant without additional factors triggering GFR decline and hemodialysis was introduced again 15 months after his discharge.

## 3. Discussion

In anti-GBM disease, the early, aggressive immunosuppressive therapy and plasmapheresis before progression of severe glomerular damage are essential for recovery of renal function [1]. However, there are some factors that cause delaying the treatment. One is the differentiation of infection from active anti-GBM disease and another is a presence of predictors for dialysis-dependence. As for the former factor, it is reported that the majority of patients at presentation had fever with respiratory tract infections, which needs further investigation to reveal their pathophysiological role in anti-GBM disease [10]. However, it is noteworthy that elevation of PCT, which is thought to be indicator for infection, together with elevation of CRP is often seen in patients with anti-GBM disease in the absence of infection [8] like in patients with other autoimmune diseases [7]. The mechanisms of elevation of PCT are not well known, but it is suggested that high PCT values in Goodpasture's syndrome might rather reflect severe organ damage of lungs and/or kidneys compared to infection [8]. Thus, physicians can start immunosuppressive therapy immediately after screening of the infection. On the other hand, it is reported that predictors for dialysis-dependence in anti-GBM disease include level of SCr ≥ 600 *μ*mol/L, oliguria, requiring dialysis at presentation, and more than 80–100% of crescent formation [5, 6, 11–13]. Levy et al. [5] reported that, among anti-GBM disease patients treated with immunosuppressants and plasma exchange, patients presenting dialysis-dependence showed only 8% renal survival at 1 year. The aggressive therapy might bring just the increased risk of immunosuppression compared to the likelihood of benefit to the patients with these predictors but without pulmonary hemorrhage. It is also reported that anti-GBM antibody titer could decrease spontaneously with time after introducing dialysis [13] and relapse of anti-GBM disease is rare [1]. KDIGO clinical practice guideline for glomerulonephritis [9] stated that “we recommend initiating immunosuppression with cyclophosphamide and corticosteroids plus plasmapheresis in all patients with anti-GBM glomerulonephritis except those who are dialysis-dependent at presentation and have 100% crescents in an adequate biopsy sample, and do not have pulmonary hemorrhage” and that “start treatment for anti-GBM glomerulonephritis without delay once the diagnosis is confirmed. If the diagnosis is highly suspected, it would be appropriate to begin high-dose corticosteroids and plasmapheresis while waiting for confirmation.”

KDIGO guideline does not recommend aggressive therapy to the patients who are dialysis-dependent at presentation. However, in accordance with this guideline half of the patients will not receive immunosuppressive therapy plus plasmapheresis because approximately half of the patients require hemodialysis at the point of initial presentation in large series [5]. Our patient could discontinue hemodialysis despite having all the reported predictors for dialysis-dependence in anti-GBM disease. Kidney biopsy for definitive diagnosis of anti-GBM disease has a potential risk for hemorrhage, which causes difficulty and delay to initiate hemodialysis when necessary. Therefore, to not miss the therapeutic windows, it is worth challenging the treatment with high-dose corticosteroids plus plasmapheresis immediately after screening of the infection before kidney biopsy irrespective of any predictors for dialysis-dependence if the diagnosis of anti-GBM disease is highly suspected.

The effectiveness of plasmapheresis for improving renal function in anti-GBM disease has been reported [14, 15]. Johnson et al. demonstrated a much more rapid fall in circulating anti-GBM antibodies and improved kidney function in patients receiving plasmapheresis when compared with immunosuppressant alone [14]. Plasma exchange of 4 L of plasma for 5% albumin was commonly performed daily for 14 days or until the circulating anti-GBM antibodies were no longer detected [16]. We used fresh frozen plasma as the substitution for plasma for fear of pulmonary hemorrhage. Due to the limitation of medical insurance in Japan, plasmapheresis could not be performed in our patient until anti-GBM antibody disappeared. Double-filtration plasmapheresis for selectively removing the immunoglobulin fraction from serum [17] combined with immunosuppressive therapy was reported to be effective and a good removal efficacy of anti-GBM antibody in one case with Goodpasture's syndrome [18]. Small series using immunoadsorption for the removal of pathogenic autoantibody in anti-GBM disease [19] documented comparable outcomes when compared with plasma exchange therapy [20, 21].

The 2nd kidney biopsy showed the progression of irreversible glomerular damage 7 weeks after the therapy. The difference of immunofluorescence microscopy result between two kidney biopsies was shown in Table 1. Unfortunately, renal specimen for electron microscopy included only sclerotic glomerulus in the second biopsy. The deposited immunoglobulin in our case was of IgG1 subclass, though the predominant IgG subclass was reported to be IgG3 [22]. Linear deposition of *κ* and *λ* light chains, with *λ* staining being more intense than *κ*, was found in the first biopsy, but linear deposition of only *κ* light chain was found in the second biopsy. The mechanism of this difference is not known, but the modification of the antigenicity of the deposited antibodies by either natural course or the treatment might have contributed to the result. Although our patient started the therapy only 9 days after initial symptoms, he had to undergo permanent hemodialysis 15 months after the discontinuation of dialysis. Since a regimen of combination therapy using corticosteroid, cyclophosphamide, and plasmapheresis is used as standard treatment in patients with anti-GBM disease [9], additional treatment with cyclophosphamide with modification of dosage and timing of hemodialysis [23] might have been more effective in preventing progression of glomerular damage in our patient.

In summary, physicians should keep in mind that active anti-GBM disease can show high PCT value in the absence of infection. To pursue recovery of renal function, it is practically important to immediately challenge starting and continuing high-dose corticosteroid therapy plus plasmapheresis in the patients with highly suspected anti-GBM disease despite having predictors for dialysis-dependence. In addition to introduction of every possible early treatment, the treatment to inhibit glomerular damage should be established especially in patients undergoing dialysis therapy.

## Figures and Tables

**Table 1:** The immunofluorescent findings of 1st and 2nd kidney biopsies.

	IgA	IgG	IgM	C1q	C3	*κ*	*λ*	IgG1	IgG2	IgG3	IgG4
1st	−	++	−	−	±	+	++	++	−	−	−
2nd	−	++	−	−	±	++	−	++	−	−	−

*κ*: light chain *κ*; *λ*: light chain *λ*; −: negative; ±: faint staining; +: weak staining in a linear pattern; ++: strong staining in a linear pattern. The tubular basement membrane was negative for all immunoreactants examined.
